# Microglia-mediated drug substance transfer promotes chemoresistance in brain tumors: insights from an in vitro co-culture model using GCV/Tk prodrug system

**DOI:** 10.1186/s12935-024-03213-8

**Published:** 2024-01-18

**Authors:** Sheng-Yan Wu, Wen-Jui Yu, Ting-Yi Chien, Yu-An Ren, Chi-Shuo Chen, Chi-Shiun Chiang

**Affiliations:** 1 Department of Biomedical Engineering and Environmental Sciences, National Tsing Hua University, Hsinchu, 30013 Taiwan; 2 Institute of Nuclear Engineering and Science, National Tsing Hua University, Hsinchu, 30013 Taiwan; 3 Frontier Research Center On Fundamental and Applied Sciences of Matters, National Tsing Hua University, Hsinchu, 30013 Taiwan

**Keywords:** Microglia, Glioma, Tumor microenvironment, Chemoresistance

## Abstract

**Background:**

It is well known that tumor-associated macrophages (TAMs) play essential roles in brain tumor resistance to chemotherapy. However, the detailed mechanisms of how TAMs are involved in brain tumor resistance are still unclear and lack a suitable analysis model.

**Methods:**

A BV2 microglial cells with ALTS1C1 astrocytoma cells in vitro co-culture system was used to mimic the microglia dominating tumor stroma in the tumor invasion microenvironment and explore the interaction between microglia and brain tumor cells.

**Results:**

Our result suggested that microglia could form colonies with glioma cells under high-density culturing conditions and protect glioma cells from apoptosis induced by chemotherapeutic drugs. Moreover, this study demonstrates that microglia could hijack drug substances from the glioma cells and reduce the drug intensity of ALTS1C1 via direct contact. Inhibition of gap junction protein prevented microglial-glioma colony formation and microglia-mediated chemoresistance.

**Conclusions:**

This study provides novel insights into how glioma cells acquire chemoresistance via microglia-mediated drug substance transferring, providing a new option for treating chemo-resistant brain tumors.

**Supplementary Information:**

The online version contains supplementary material available at 10.1186/s12935-024-03213-8.

## Background

Glioma is the most common primary malignant tumor in the central nervous system (CNS), with grade III anaplastic astrocytoma and grade IV glioblastoma (GBM) remaining one of the most aggressive and lethal cancers [1]. For newly diagnosed patients, gross tumor resection was followed by irradiation and concomitant administration of temozolomide (TMZ) or other adjuvant drugs, including procarbazine, vincristine, and cisplatin [2]. Combining TMZ with other drugs to stop glioma cell proliferation and target multiple pathogenic pathways shows promise [3]. For example, cisplatin, a platinum-based anticancer drug, has the potential to treat various tumors, including glioma [4]. The ability of platinum compounds to decrease O^6^-alkylguanine DNA-alkyl transferase (AGAT) activity had brought clinical trials to examine the potential of TMZ-cisplatin combination for recurrent glioma patients at early time. An 34% progression-free survival rate at 6 months (PFS-6) was reported [5]. However, a recent report indicated that the combination of cytotoxic drugs had little improvement in clinical overall survial. The PFS-6 varied from 18–48% for naïve TMZ patients, 8–58% for TMZ retreated patients, and 6–43% for re-irradiated patients [6]. Despite these intensive care and treatments, the median survival time for high-grade (WHO classification; grade >III) glioma (HGG) patients was only 18 months, while the 5-year survival rate for GBM patients was only 4 to 7% [7, 8]. The limited therapeutic efficacy is mainly due to the high relapse rate of HGG, indicating tumor resistance to the therapy [3]. Hence, developing novel strategies for overcoming resistance and understanding the resistance mechanism is key to translating knowledge into clinical practice against aggressive glioma.

The focus of tumor resistance has shifted from the intrinsic tumor cell to the tumor microenvironment (TME) [9–11]. The TME is heterogeneous and consists of stromal cells, immune cells, endothelial cells, and an extracellular matrix [12]. One theory is that stromal cells or tumor-associated macrophages (TAMs) could protect tumor cells from drug-induced apoptosis via secreting interleukin-6 (IL-6) to activate signal transducer and activator of transcription 3 (STAT3) pathway of tumor cells [13, 14]. Other studies suggest that cancer cells were protected from excessive reactive oxygen species (ROS) via direct contact with stromal cells for mitochondria transferring or through gap junction moving [15, 16]. These studies suggest that cells within TME are vital in affecting tumoral resistance to therapies.

The TME of glioma is remarkably heterogeneous. Recent single-cell transcriptional analysis of the murine GBM model revealed more than 30 clustered cell types, including 17 clusters of CD45^−^ non-immune cells and 20 clusters of CD45^+^ immune cells [17]. Similarly, another study in human glioma samples revealed 12 clusters of cells with unique gene expression patterns [18]. Among these cells, microglia were the most abundant cells in the TME of the glioma, accounting for over 60% of the immune cells [19]. Microglia derive from the yolk sac progenitors and become the naïve aborigines in the CNS region. They are highly dynamic and constantly respond to variations in the CNS microenvironment to achieve tissue homeostasis. Thus, microglia would be the first cells to respond to the appearance of tumor cells in the CNS [20, 21]. However, it is difficult to solely investigate their roles in the tumor progression due to their similarity with infiltrated macrophages even with recent lineage-tracing and single-cell sequence identifying core microglia signature markers, TMEM119, P2ry12, and SALL1 [22, 23]. Microglia phenotypically change to a proliferative state once they have encountered enough proximity to cancer cells. They produce cytokines like IL-1 and IL-18 to promote tumor progression [24, 25]. However, their roles in developing tumor chemoresistance are still lacking. Our previous in vivo animal study demonstrated that astrocytoma was highly infiltrated with F4/80^+^ macrophages/microglia [26]. We found that these F4/80^+^ macrophages/microglia were critical in increasing tumor mean vessel density, and promoting tumor recurrence following drug treatment [27]. However, it did not distinguish macrophages from microglia in this in vivo model, and the detailed mechanism of how microglia affect brain tumor resistance to chemotherapy is still unclear.

In this study, we established a co-cultured system of the astrocytoma cell line, ALTS1C1, and microglia cell line, BV2, in vitro to mimic the microglia-rich TME of the glioma. Our results demonstrated that a colony structure could rapidly form once the microglia and glioma cells had enough cell–cell contact. The colony structure could further protect cells from therapeutic drug-induced apoptosis. Moreover, we found that microglia cells could hijack the drug substance from the glioma cells through direct contact, thus decreasing the drug intensity of the cancer cells. This protective phenomenon was mediated by gap junction protein. Inhibition of the gap junction protein disrupted microglia-glioma colony formation and wrinkled the microglial protective effect on glioma cells against the therapeutic drugs.

## Material and methods

### Cell lines culture

Murine astrocytoma cell line, ALTS1C1 (T8239, Applied Biological Materials, Richmond, Canada, or BCRC60582, Hsinchu, Taiwan), and a stable clone of ALTS1C1-Tk was previously established in our lab [26, 27]. GL261, a murine glioma cell line, was kindly provided by Dr. Newcomb’s lab at New York University Medical Center. B16-F0, a murine melanoma cell line, was purchased from ATCC (CRL-6322, Manassas, VA, USA). ALTS1C1-GFP and GL261-GFP cell line was created by lentiviral infection of GFP-expressing vector into the ALTS1C1 cells, and GL261 cells respectively, ALTS1C1-GFP-Tk cell line was created by transfection of herpes simplex virus thymidine kinase (HSV-Tk) gene into the ATLS1C1-GFP cell line. The GL261-Tk cell line was created by transfection of herpes simplex virus thymidine kinase (HSV-Tk) gene into the GL261 cell line. UN-KC-6141, a pancreatic adenocarcinoma cell line, was kindly provided by Prof. Surinder K. Batra at the University of Nebraska Medical Center [28]. The immortalized murine microglia cell line, BV2 (ATL03001, ICLC, Genova), was used to represent microglia [29]. Cells were maintained in 37 °C, 5% CO_2_ humidified air atmosphere with Dulbecco’s modified Eagle’s medium (DMEM; Gibco®, 12100046, Grand Island, NY, USA). The DMEM medium contained 10% fetal bovine serum (FBS; Gibco®, 16000044) and 1% penicillin–streptomycin (PS; Gibco®, 15140122). Mycoplasma contamination before the experiment was examined with the EZ-PCR™ Mycoplasma Detection Kit (Biological Industries, 20-700-20, Beit Haemek, Israel). Cells were co-cultured in a 1:1 ratio (if not mentioned) following the indicated seeding density of 0.8 (low density), 1.6 (medium density), and 3.2 (high density) × 10^3^ cells/mm^2^ for 24 h. Then, the images were taken via an inverted microscope (ZEISS, Axiovert 40 CFL, Gottingen, Germany). Colony size was calculated by Image-pro plus 6.0 (Media Cybernetics, Inc., MD, USA).

### Orthotopic brain tumor injection

The orthotopic brain tumor model was established as previously described [26]. Brifly, mice were anesthesia and epilate of hair. 1 × 10^5^ of ALTS1C1-GFP or GL261-GFP cells in a 2 μl volume were intracranial injected into the brain. The injection site was drilled and located 1.0 mm posterior to the bregma and 2.0 mm lateral to the midline, with a depth of 2.5 mm. After the injection, the opening was sealed with bone wax (ETHICON, W810, Somerville, NJ, USA). After sealing, the mice’s skin was sutured with two stitches. All animal procedures followed the guidance of the Institutional Animal Care and Use Committee (IACUC) of the National Tsing-Hua University (IACUC approval No. 107042).

### Brain tissue collection and immunofluorescence staining

18 days after the tumor injection, mice were sacrificed, and cardio-vascular perfusion was performed with 4% paraformaldehyde (Sigma, 16005) solution in PBS, pH 7.4. The brain tumor tissue was carefully removed and embedded into the OCT (Optimal Cutting Temperature) compound (Sakura Finetek, 4583, Torrance, CA, USA). The tissues were immediately stored in the − 80 °C refrigerator. Frozen tissues were sectioned 10 μm with a cryo-stat (Leica, CM1850, IL, USA). For immunofluorescence staining. The tissue section slides were fixed with methanol and permeabilized using 0.05% Tween-20 (SIGMA, p1379-500ML). Subsequently, the slides were mounted with a blocking buffer (containing 4% FBS and 1% goat serum in 1 × PBS) for 1 h. The primary antibody of purified rabbit anti-mouse TMEM119 (1:200, Abcam, 209,064, Cambridge, UK) was stained overnight at 4 °C. A secondary antibody, Alexa Fluor 594 goat anti-rabbit (1:200 Thermo Fisher Scientific, A11012) was applied the other day for 1 h at room temperature. The whole tumor image was captured with a laser scanning confocal (ZEISS, LSM-780), and the image with a larger view was captured with a fluorescence microscope (ZEISS, Axioskov 40).

### Condition medium preparation

ALTS1C1, BV2 cells, or co-culture of ALTS1C1 and BV2 were cultured in the highest density condition for 24 h, then the supernatant of each medium was collected without the cells. The supernatant was subsequently mixed with the fresh medium in a 1:1 ratio as the conditioned medium. ALTS1C1 was cultured in the conditioned medium at the highest seeding density for 24 h.

### MTT Cytotoxicity assay

5 × 10^3^ cells were seeded in the 96-well plate overnight; the next day, diluted cisplatin (Fresenius Kabi, Kemoplat, Solan, India) or ganciclovir (GCV; Sigma, G2536, MO, USA) was added to the medium and incubated at 37 °C, 5% CO_2_ for 72 h. After incubation, 250 μg of 3-(4,5-Dimethylthiazol-2-yl)-2,5-diphenyltetrazolium bromide (MTT, Sigma, SI-5655) was added into each well for 3 h and measured by the plate reader (TECAN, Infinity® 200 PRO, Männedorf, Switzerland).

### Co-culture and chemo-apoptosis experiments

Cells were co-cultured at the highest density for 24 h, then 10 μg/ml of GCV or together with various concentrations (62.5, 125, and 250 μM) of carbenoxolone (CBX; C4790, Sigma) was administered in the medium for the indicated time of incubation. After incubation, cells were collected for apoptotic staining via the Apoptosis detection kit (BD Pharmingen, 556547, San Jose, CA, USA). According to the manufacturer’s protocol, cells were stained with Annexin V-FITC and propidium (PI). To distinguish BV2 microglia from ALTS1C1-Tk or GL261-Tk cells, a pan-leukocyte marker CD45 (BD Pharmingen, 552848) was applied. After staining, cells were processed and analyzed via flow cytometry (BD Biosciences, FACSCanto™, San Jose, CA, USA). The flow cytometry gating strategy is depicted in Additional file 1: Figure S2b.

### Cell transfection

The cell transfection was performed with the Effectene Transfection Reagent kit (QIAGEN Biotechnology Inc, 1054250, Mainz, Germany). HSV-Tk gene was transfected into the ATLS1C1-GFP or GL261 cells and the protocols were previously described [27]. After the transfection, 1.5 mg/ml G418 (Sigma, A1720) was applied to select stably transfected ALTS1C1-GFP-Tk and GL261-Tk cells.

### Cell immunofluorescence staining

Cells were co-cultured at the highest density on the 4-well chamber slide (LAB-TEK®, 154526, Roskilde, Denmark). After incubation, cells were fixed with 4% paraformaldehyde for 10 min and blocked with 5% FBS and 1% goat serum (GS; Gibco®, 16210-064) for 1 h. Then, the primary antibodies, purified rat anti-mouse CD11b (1:200, BD Pharmingen, 550282), and purified rabbit anti-mouse Caspase-3 (1:200, BD Pharmingen, 559565) were added and incubated at 4 °C overnight. Secondary antibodies, Alexa Fluor 488 goat anti-rat (1:200 Thermo Fisher Scientific, A11006, Waltham MA, USA) and Alexa Fluor 594 goat anti-rabbit (1: 200 Thermo Fisher Scientific, A11012), were applied on the next day for one hour at room temperature. Three images were taken via the fluorescence microscope in each experiment and were repeated three times, statistics were quantified with Image-pro plus 6.0 software by gating the caspase-3^+^ and GFP^+^ double positive area divided by all GFP^+^ area. For confocal imaging of co-culture, BV2 was stained with membrane dye Fast-Dil (Thermo Fisher Scientific, D7756) for 15 min before co-culturing with ALTS1C1-GFP cells in a 12-Well culture dish. The images were taken by the laser scanning confocal (ZEISS, LSM-780) after 24 h of co-culturing.

### Cell colony CD133 staining

The microglia-glioma colonies were collected via viciously shaking in 1 to 3 PBS diluted Accutase (Innovative Cell Technologies, Inc., AT104, San Diego, CA, USA) for 2 min. Then, the collected cells were resuspended in the blocking buffer with 1% GS and 0.2% FC block (BD Pharmingen, 553142) for 30 min on ice. After blocking, cells were stained with fluorescence-conjugated antibodies against CD45, CD133 (Invitrogen, 17-1331-81, CA, USA), and the isotype control of CD133 (Invitrogen, 17-4301-82) for 45 min before flow cytometry analysis.

### Cell cycle analysis

Cells were seeded at low-density or high-density for 24 h. After 24 h, cells were harvested, and microglia cells were further separated from glioma cells by CD45 MicroBeads (Miltenyi, 130-052-301, Bergisch Gladbach, Germany). After separation, cells were stained with the PI solution containing 40 μg/ml Propidium Iodide (PI; Sigma,

P4170), 200 μg/ml RNase (Thermo Fisher Scientific, 120091021, Carlsbad, CA, USA), and 0.2% Triton X-100 (Sigma, T9284) for 15 min at room temperature before flow cytometry analysis. The cell cycle data were analyzed by FlowJo™ software (BD Biosciences, FlowJo™ v10).

### Dye/drug substance transfer experiment

Cells were pre-treated with 10 μg/ml doxorubicin (Dox; Pfizer, ADRIAMYCIN™, Bentley, Australia) or 2.5 μg/ml Fast-DiO (Thermo Fisher Scientific, D3898) for 30 min. After incubation, cells were washed with 1X PBS three times before the co-culture experiments. Then, treated cells were co-cultured with a 1:1 ratio with the untreated cells at the high-density condition for 4, 12, or 24 h. After the co-culture, cells were collected for flow cytometry analysis. The doxorubicin or Fast-DiO positive cells were gated and analyzed by FlowJo™. The median fluorescence intensity (MFI) ratio was calculated with the variation of the median fluorescence intensity of the treated cells to the untreated control cells. The time-lapse image of the drug transportation process was captured by the automated inverted microscope (Ti-Eclipse, Nikon, Tokyo, Japan) and the camera (ORCA-Flash4.0, Hamamatsu, Japan) under the onstage incubator for the general culture conditions.

### Statistics

The significance analysis was performed by the Prism software 8.0 (GraphPad, San Diego, CA, USA) with Student’s *t*-tests. The results were considered statistically significant if the P value was below 0.05.

## Results

### Effect of microglia on brain tumor cells

**Fig. 1:**
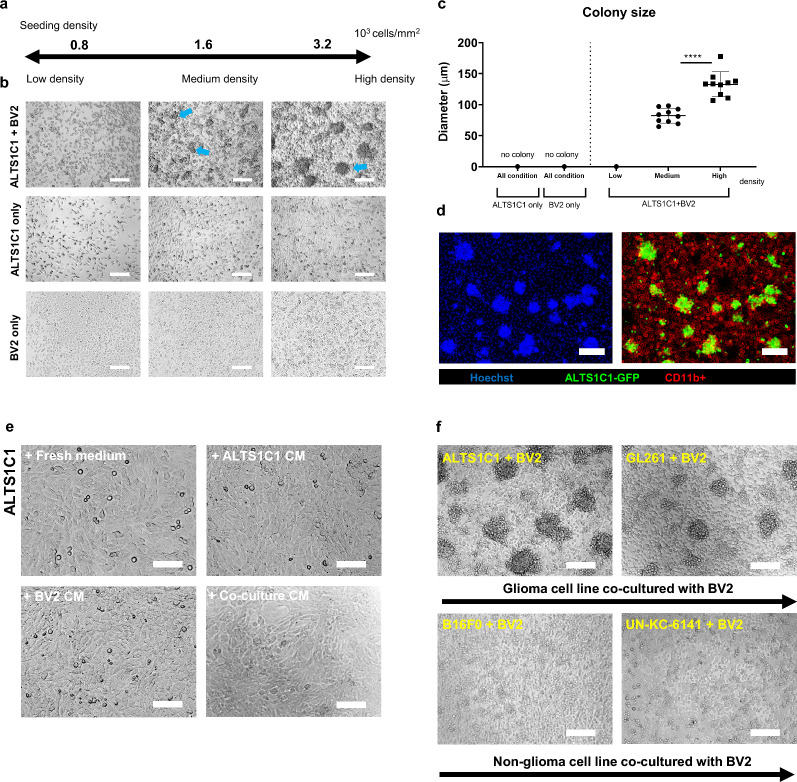
Effect of microglia on brain tumor cells. **a** The diagram of cell co-culture experiment designation indicates high-density (3.2 × 10^3^/mm^2^), medium-density(1.6 × 10^3^/mm^2^), and low-density(0.8 × 10^3^/mm^2^) seeding conditions. **b** The images of co-culture on different seeding intensities after 24 h of incubation. The blue arrow indicates the colony (a cluster of diameter > 50 μm). Scale bar = 200 μm. **c** Statistics of the average colony diameter among different conditions. **d** The immunofluorescence staining of ALTS1C1-GFP (Shown in green) co-culture with BV2 for 24 h. The nucleus was stained with Hoechst (Left picture, shown in Blue), and the cells were stained with myeloid cell marker CD11b (Right, shown in red). Scale bar = 200 μm. **e** The images of ALTS1C1 were cultured in different condition mediums for 24 h of incubation. Scale bar = 100 μm. **f** The images of different cancerous cell lines co-cultured with BV2 for 24 h. Scale bar = 200 μm. A two-tailed unpaired t-test was used to compare every two groups. ****P < 0.0001

Our previous study on brain tumors suggested that F4/80^+^ microglia/macrophages played essential roles in increasing tumor mean vessel density and recurrence following therapy [27]. However, the detailed resistance mechanisms of how macrophages/microglia affect tumor cells were unknown. A recent study demonstrated that in the TME of glioma, resident microglia had distinct roles from infiltrating macrophages [19]. To explore the TME in the orthotopic murine models of ALTS1C1-GFP and GL261-GFP, immunofluorescence staining of microglia marker TMEM119 was applied. The results suggested the whole ALTS1C1-GFP and GL261-GFP tumors (depicted in green) were intensively surrounded by the TMEM119^+^ cells (shown in red) (Additional file 1: Figure S1a). Closed contact of TMEM119^+^ cells and the GFP^+^ tumor cells were also observed (Additional file 1: Figure S1b). To further explore the interactions between tumor cells and microglia, a dual cell culture system in vitro was employed to simulate the microglia-rich tumor microenvironment. The astrocytoma cell line, ALTS1C1, was co-culturing with BV2 (a murine microglial cell line) in equivalent cell numbers for 24 h. The seeding density of the co-culturing was evaluated since the cell proximity (< 10 μm) was reported to play a pivotal role in effective direct cell–cell contact [30]. Cell–cell proximity was increased from 21 μm to 1 μm by varying the seeding density from 0.8 to 3.2 × 10^3^ cells per square millimeter, corresponding to low, medium, and high-density conditions (Fig. 1a, Additional file 1: Figure S1c). Surprisingly, ALTS1C1 was found to form colonies (diameter > 50 μm, blue arrow in Fig. 1b) with BV2 cells under medium and high-density conditions. The diameter of the colony significantly increased from 82 to 133 μm as the density increased from medium to high density, while no colony was observed in low-density criteria. Meanwhile, mono-cell culture couldn’t form colonies in any condition (Fig. 1b, c). To further understand the colony composition, immunofluorescence of Hoechst (nucleus staining) and the myeloid cell surface maker CD11b were applied to the ALTS1C1-GFP and BV2 co-culture. The image (Fig. 1d) demonstrated that the major cells inside the colony were the GFP tumor cells while the CD11b^+^ BV2 cells (Red color) were in the peripheral. The confocal image (Additional file 1: Figure S1d) further suggested that ALTS1C1-GFP cells were piling up inside the colony. The 3D larger view shows that the majority are GPF tumor cells in the colony. However, a few BV2 cells (stained with membrane dye, Fast-Dil, shown in red) could also be observed inside the colony. To validate whether secreted factors mediated the colony formation, the conditioned medium from ALTS1C1, BV2, or co-culture condition was applied to ALTS1C1 cells. The result showed that no colonies were seen in the presence of either ALTS1C1, BV2, or co-culture condition mediums after 24 h (Fig. 1e). Also, the condition medium from the co-culture neither affected the process of colony formation (Additional file 1: Figure S1e). We co-cultured other types of tumor cells with BV2 to corroborate if the microglia-tumor colony embodiment was cell type-specific. The result demonstrated that the BV2-associated colony was seen not only in ALTS1C1 but in the other common murine glioma cell line, GL261. However, the melanoma cell line, B16F0, or pancreatic tumor cell line, UN-KC-6141, could not colonize in the same co-culturing condition (Fig. 1f). These results concluded that brain tumor cells could be colonized in the presence of BV2 microglia under high-density seeding conditions, and this special spheroid formation required direct contact of tumor cells with microglia.

### Chemo-resistance of glioma cells after co-culturing with BV2

**Fig. 2:**
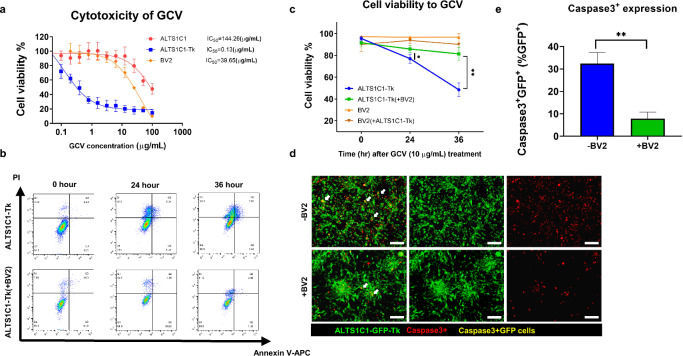
Chemo-resistance of ALTS1C1 after co-culturing with BV2. **a** The MTT cytotoxicity assay of the pro-drug GCV on ALTS1C1, ALTS1C1-Tk, and BV2 cell lines for 72 h of incubation. **b** Representative dot plot FACs images of the co-culture chemo-apoptosis assay of 10 μg/mL pro-drug GCV on ALTS1C1-Tk only or ALTS1C1-Tk (+BV2) for 0, 24, 36 h. **c** Quantification of the chemo-apoptosis assay. N ≧ 3. **d** Representative caspase-3 staining images of ALTS1C1-GFP-Tk, ALTS1C1-GFP-Tk co-cultured with BV2 treated with 10 μg/mL GCV for 24 h. Scale bar = 200 μm. **e** Quantification data of the caspase-3 staining. N = 3. A two-tailed unpaired t-test was used to compare every two groups. *P < 0.05, **P < 0.01

Various cancer cells were found to form spheroids under serum-free or ultra-low culture conditions, and these spheroids were more resistant to chemo-drugs than mono-culturing [31, 32]. To further evaluate whether the BV2-mediated colony formation could protect the tumor cells from chemotherapy, a follow-up cytotoxicity system was established in high density (cell–cell ratio 1:1) seeding condition (if not mentioned). First, the alkylating chemotherapy drug cisplatin was chosen to investigate whether it exhibits toxic effects on microglia, as previously reported with TMZ [33, 34]. The result demonstrated that it caused similar cell cytotoxicity to both the ALTS1C1 and BV2 cells (IC_50_ = 2.56 and 2.81 μg/ml, respectively), thus making it difficult to target the tumor cells specifically (Additional file 1: Figure S2a). We then applied the Herpes simplex virus thymidine kinase /pro-drug ganciclovir (HSV-Tk/GCV) system to target tumor cells specifically. The suicide gene HSV-Tk was transfected into the ALTS1C1 cells as previously described [27]. A stably transfected cell line, ALTS1C1-Tk, was used for a specific chemo-drug targeting test. The MTT assay results demonstrated that the parental ALTS1C1 and BV2 cells were less sensitive to the toxicity of pro-drug GCV, as the IC_50_ was 144.26 and 39.65 μg/ml, respectively. However, the IC_50_ of GCV to ALTS1C1-Tk was only 0.13 μg/ml, almost 300 times more sensitive than the BV2 cells (Fig. 2a). A concentration of 10 μg/ml GCV that resulted in about 80% survival of BV2 cells and 20% survival of ALTS1C1-Tk cells was chosen for further experiments. ALTS1C1-Tk cells were co-cultured with BV2 cells for 24 h, and then 10 μg/ml GCV was added to treat the cells for 24 and 36 h. Cells were collected for flow cytometry, and the gating strategy is depicted in Additional file 1: Figure S2b. The population of single cells was first gated, and a pan-leukocyte marker CD45 was utilized to separate the BV2 from ALTS1C1-Tk cells. The un-apoptosis lived cells were determined by Annexin V/PI staining (Annexin V^−^PI^−^ cells). As shown in Fig. 2, the survival rate of ALTS1C1-Tk cells alone following the GCV administration was time-dependent, with 77.5 and 48.5% lived cells after 24 and 36 h, respectively, of treatment (Fig. 2a, b). However, ALTS1C1-Tk co-culturing with BV2 cells exhibited a significantly higher percentage of lived cells at 24 h (85.9 vs. 77.5%) and 36 h (81.5 vs. 48.5%) after the GCV treatment, demonstrating the protective effect of BV2 on ALTS1C1-Tk cells against the cytotoxicity of GCV. Meanwhile, the BV2 or the BV2 co-cultured with ALTS1C1-Tk displayed no significant variation of the cell viability throughout the treatment, constantly retaining above 90% survival (Fig. 2b, c, Additional file 1: Figure S2c). To further confirm the apoptosis result, the immunofluorescence staining of another early apoptotic marker, Caspase-3, was applied. Here, we further transfected the HSV-Tk gene into the ALTS1C1-GFP cells; the stable clone of the cell line was named ALTS1C1-GFP-Tk. The green fluorescence of this cell line enabled us to visualize tumor cells while retaining similar specific cytotoxicity to the pro-drug GCV (Additional file 1: Figure S2d). The immunofluorescence result illustrated many GFP^+^ cells with the Caspase-3^+^ expression (yellow color, white arrow) in the ALTS1C1-GFP-Tk, indicating an early sign of apoptosis after 24 h of GCV treatment. However, when ALTS1C1-GFP-Tk cells were co-cultured with the BV2 cells, the Caspase-3^+^ expression on GFP^+^ cells significantly dropped from 32.4% to 7.8% (Fig. 2e). On the other hand, BV2 only barely had Caspase-3^+^ expression (Additional file 1: Figure S2e). Together, these results indicated that BV2 might have increased the chemoresistance of the ALTS1C1 cells, preventing them from drug-induced apoptosis.

The previous data has shown that the GL261 glioma cell line, in addition to ALTS1C1, can also form a colony structure when co-cultured with BV2 cells (Fig. 1e). This suggests that BV2 cells might offer a protective effect similar to ALTS1C1 on GL261 cells, extending beyond tumor heterogeneity. To investigate this further, additional experiments using the GL261-Tk cell line were conducted. The results revealed that the response of GL261-Tk cells to GCV treatment was time-dependent, with cell survival rates of 87.6%, 85.9%, and 74.3% after 36, 48, and 60 h of exposure to 10 μg/ml GCV, respectively. However, when GL261-Tk cells were co-cultured with BV2 cells, their survival rate was significantly higher at all time points: 90.8% versus 87.6% at 36 h, 93.0% versus 85.9% at 48 h, and 89.4% versus 74.3% at 60 h after GCV treatment. These findings demonstrated the protective effect of BV2 co-culture on GL261-Tk cells against GCV-induced cytotoxicity (Additional file 1: Figure S3a, b). The above findings suggested that BV2 cells could provide protection not only to ALTS1C1 cells but also to GL261 cells against drug-induced apoptosis, indicating a universal effect of BV2 across different glioma cell lines.

**Fig. 3:**
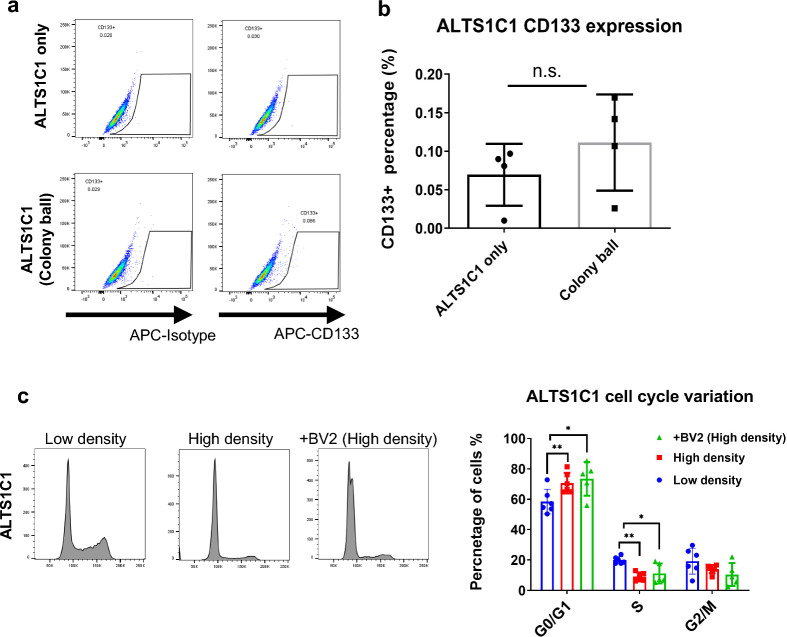
The effect of BV2 co-culturing on ALTS1C1 cells. **a** Representative flow dot plots of CD133, isotype staining on ALTS1C1 or the colony from the ALTS1C1 and BV2 after co-culturing for 24 h. **b** The quantification of flow analysis on CD133. **c** The cell cycle analysis on ALTS1C1 of different culturing conditions (Low density, High density, and co-cultured with BV2 on high density). A two-tailed unpaired t-test was used to compare every two groups. *P < 0.05, **P < 0.01

Numerous cancer cells possessed stem-cell-like properties and could form colony structures. It has been proposed that the chemoresistance of cancer stem cells is due to self-renewal and staying quiescent [35]. In light of this, we then asked whether the co-cultured astrocytoma and microglia colonies had stem-cell-like characteristics to exile the drug protection effect. A specific cluster differentiation stem cell marker, CD133, was applied to stain the colonies after co-culturing for 24 h. The results suggested that the percentage of the CD133^+^ cells of ALTS1C1 in the colonies was meager and not significantly increased compared to the ALTS1C1 only (Fig. 3a, b). However, the cell cycle analysis showed that high-density cell seeding conditions constrained the ALTS1C1 cells in the G0/G1 phase as the percentage of G0/G1 cells significantly increased from 58.5 (low-density) to 70.6 (high-density) and 73.5 (co-cultured high-density condition). While the percentage of S-phase cells significantly dropped from 19.9 to 9.1 and 11.3 (co-cultured high-density condition) (Fig. 3c). The high-density condition also constrained BV2 in the G0/G1 phase as the percentage of cells in G0/G1 significantly increased while cells in S, G2/M phase decreased (Additional file 1: Figure S4). The above cell cycle experiment data revealed that the cells tended to stay quiescence in the colonies. However, the previous survival data demonstrated that ALTS1C1-Tk cells alone in high-density conditions are still sensitive to the cytotoxicity of the GCV, indicating that the presence of BV2 was crucial for drug resistance. The above data demonstrated that the drug resistance feature of ALTS1C1 in this model might come from BV2 rather than the stem-cell-like or quiescent characteristics of cells in spheroids.

### BV2 hijack drugs from ALTS1C1 through direct contact

**Fig. 4:**
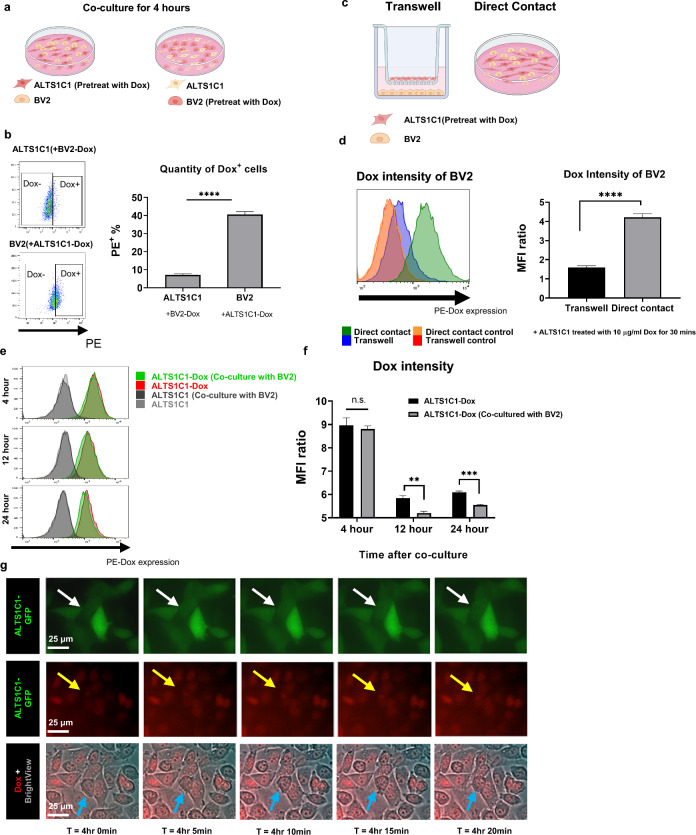
BV2 hijacks drugs from ALTS1C1 through direct contact. **a** The experimental scheme on the Dox transfer test between the ALTS1C1 and BV2 cells. **b** Representative flow images of PE-positive cell percentages indicating Dox-positive cells (Left), and the quantification of Dox-positive cells after co-culturing for 4 h (Right). **c** In-direct transfer experiment scheme. **d** Representative flow intensity images of in-direct transfer experiment (Left), and the quantitative data of median fluorescence intensity (MFI) ratio of trans-well (in-direct) and direct contact groups. N = 3. **e** Representative flow intensity images of cells treated with Dox for 4, 12, and 24 h. **f** The quantification of median fluorescence intensity (MFI) of Dox on ALTS1C1, and ALTS1C1 (Co-cultured with BV2) for 4, 12, and 24 h. **g** Representative images of time-lapse picturing Dox transferring from ALTS1C1-GFP to BV2. Images were taken every 5 min. Scale bar = 25 μm. A two-tailed unpaired t-test was used to compare every two groups. **P < 0.01. ***P < 0.001, ****P < 0.0001

Previous survival data showed that the presence of BV2 played a vital role in affecting the chemoresistance of ALTS1C1-Tk cells. However, we noticed some Caspase-3^+^GFP^−^ cells, indicating apoptotic BV2 cells, in the Caspase-3 staining imaging (Fig. 2d). We, therefore, wondered if there existed toxic substances transported between these two cells. To address this question, membrane dye Fast-DiO was used to visualize and mimic the substance transported between cells. BV2 or ALTS1C1 cells were first stained with Fast-DiO as BV2-Fast-DiO or ALTS1C1-Fast-DiO, respectively. We co-cultured ALTS1C1 and BV2 for 12 or 24 h (Additional file 1: Figure S5a). The flow cytometry data suggested that BV2 gained FITC signal 12 h after co-culturing with ALTS1C1-Fast-DiO (28.4% and 44.8% Fast-DiO positive cells for 12 and 24 h, respectively). Interestingly, only 2.4% and 5.2% ALTS1C1 received FITC signal after co-culturing with BV2-Fast-DiO after 12 and 24 h, indicating that the major substance transfer direction might be from ALTS1C1 to BV2 cells (Additional file 1: Figure S5b, c). To further confirm whether the substance transport phenomenon happened not only for membrane dye, a fluorescence chemo-drug Doxorubicin (Dox) was utilized. ALTS1C1 or BV2 cells were first treated with 10 μg/ml Dox for 30 min, then BV2 was added for 4 h of incubation (Fig. 4a). The flow cytometry analysis revealed that 40.6% of BV2 cells gained PE signal from ALTS1C1 while only 7.1% of ALTS1C1 cells received PE signal from BV2, suggesting Dox was transferred more from ALTS1C1 to BV2 cells than from BV2 to ALTS1C1 (Fig. 4b).

To further verify whether the above drug transfer needs direct cell–cell contact or not, a cell un-permeable transwell was used. Similar to the previous setup, ALTS1C1 was pretreated with Dox for 30 min and seeded at the upper chamber of the transwell, and BV2 was seeded in the bottom for 4 h before flow cytometry analysis (Fig. 4c). The results demonstrated that BV2 in the transwell group (no direct contact) had a significantly lower MFI (median fluorescence intensity) ratio than the direct contact group (1.6 vs. 4.2) (Fig. 4d). In addition, the MFI ratio of ALTS1C1 pretreated with Dox significantly decreased by 10% after co-culturing with BV2 for 12 h, indicating the reduction of the average drug amount in ALTS1C1 cells (Fig. 4e, f). These data proved that BV2 received Dox more from the direct transfer of ALTS1C1 cells than the uptake of Dox released by ALTS1C1 cells in the medium. We further real-time assessed the direct Dox transfer between cells using time-lapse imaging. The same co-culture setting is depicted in Fig. 4a. The real-time imaging started at 4 h after the co-culture. Here, the white arrow indicated the ALTS1C1-GFP cells with a clamp of Dox signal (yellow arrow), and the cells indicated by the blue arrow without the GFP signal were considered BV2 cells. As time passed, the BV2 cells moved close to the ALTS1C1-GFP cells, and the clamp of the Dox signal was seen immediately transferred into the cytoplasm of the BV2 cells, confirming that BV2 gained the Dox from the ALTS1C1-GFP cells (Fig. 4g, Additional file 2: Video S1). The above findings suggested that the chemoresistance of ALTS1C1 cells after co-culturing with BV2 cells might result from decreased drug concentration, which was transferred to the BV2 cells through direct contact.

### The effect of gap junction on co-culture-induced chemoresistance

**Fig. 5:**
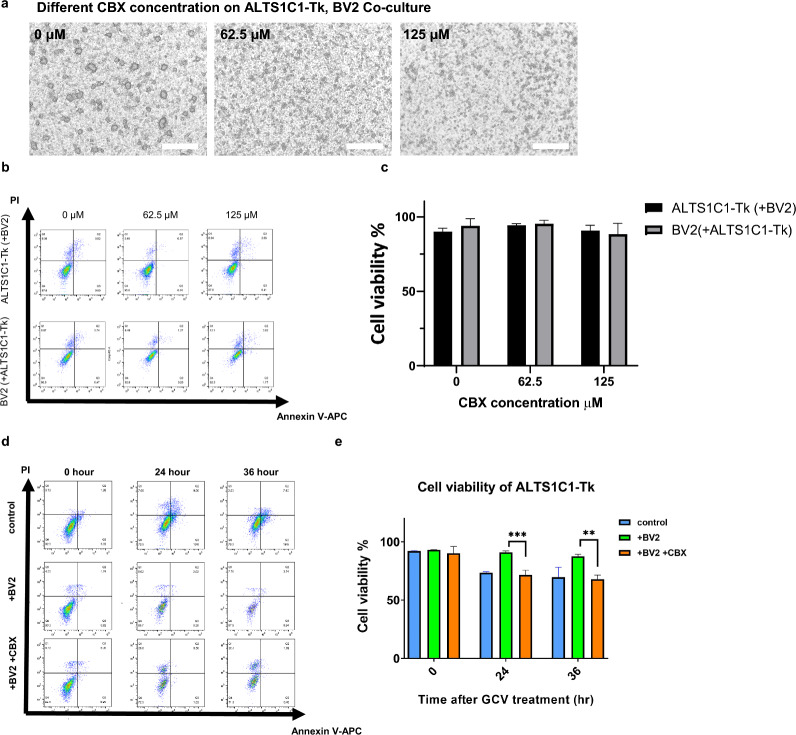
The effect of gap junction on co-culture induced chemoresistance. **a** The representative images of various concentration CBX on ALTS1C1-Tk and BV2 co-culture. Scale bar = 500 μm. **b** The representative flow images of chemo-apoptosis assay on different concentrations of CBX on ALTS1C1-Tk and BV2 co-culture. **c** statistics of chemo-apoptosis assay, N = 3. **d** Representative dot plot FACs images of the co-culture chemo-apoptosis assay of 10 μg/mL pro-drug GCV and 10 μM CBX on ALTS1C1-Tk only or BV2 co-cultured group for 0, 24, 36 h. **e** Quantification of the chemo-apoptosis assay, the protective effect has significantly weakened under CBX treatment. N = 3. A two-tailed unpaired t-test was used to compare every two groups. *P < 0.05

Previous data revealed that substances transferred between cells might be the explanation for the chemoresistance. We further investigated the roles of cell–cell transportation on microglia-mediated chemoresistance. Research has shown that gap junction proteins play essential roles in directly exchanging ions and metabolites between neighboring cells [36]. Among the gap junction proteins, connexin 43 (Cx43) was the most abundant, and its expression was related to increased permeability to chemotherapeutics [37]. This study has shown that ALTS1C1 cells could form tightly connected spheroids with the BV2 cells. To examine whether the Cx43 protein was involved in the formation of the colony structure, various concentrations of gap junction protein inhibitor CBX (carbenoxolone) were applied to the co-culture system. The results show that the integrity of the ALTS1C1-Tk-BV2 colony was disrupted in a CBX dose-dependent manner (Fig. 5a). Perceived that CBX could interrupt the colony formation, it was further curious about the CBX effect on the BV2-mediated chemoresistance. Since the cell morphology in the co-culture condition was abnormal at a concentration greater than 250 μM, we wondered if the drug would affect cell viability. The apoptosis assay was applied. The results revealed that there were no significantly increased apoptosis cells in ALTS1C1 or BV2 cells through the CBX treatment, indicating that BV2 cells and the ALTS1C1 cells were largely unaffected by different doses of CBX (Additional file 1: Figure S6a, b). Under co-cultured conditions, there were also no significant variations in the cell viability across different concentrations of CBX. The cell viability of both cell types remained consistently over 90% in the presence of 62.5 μM CBX (Fig. 5b, c). Thus, the concentration of 62.5 μM was then used for subsequent experiments. The CBX was added with the pro-drug GCV as the same experimental setup in Additional file 1: Figure S2b. The results revealed that CBX could significantly diminish the protective effect of the BV2 cells (Fig. 5c, d). The above findings indicated that the inhibition of gap junction protein interrupted the colony formation between ALTS1C1-Tk and BV2 cells and shriveled the chemo-protect effect of BV2 on ALTS1C1-Tk against the GCV treatment.

## Discussion

Resistance to chemotherapy is one of the rationales of HGG treatment failure. The TME of the brain tumor has been associated with tumor progression and resistance [38]. As one of the most abundant stromal cells in the TME, microglia have been proposed to be related to tumoral defense against chemo drugs. In this study, we established an in vitro co-culture system of astrocytoma cells and microglia cells to imitate the brain tumor TME for investigating the role of microglia in brain tumor chemoresistance. Our result demonstrated that microglia cells could render brain tumor cells to form a colony, and the colony could protect the tumor cells from apoptosis induced by the therapeutic drugs. Moreover, the real-time video indicated the drug transfer from tumor cells to the microglia. This study also demonstrated that gap junction inhibitor CBX could disrupt colony formation and relieve microglia-dependent chemoprotection. This in vitro study provides direct clues that tumor-educated microglia cells functionally act as the chemo protector of the brain tumor cells. This might explain the resistant nature of HGG in the clinical.

Many tumor–stroma interaction studies correlated the resistance of the tumor to the secreted paracrine factors or exosomes by stromal cells [39, 40]. However, these cells in the TME were also found to confer resistance through a more physical way, cell–cell contact [15]. The effective cell–cell contact distance was less than 10 μm [30]. Therefore, the cell seeding density of the co-culture system is critical. Most studies perform co-culture experiments with the seeding density range from 150 to 650 cells/mm^2^ [41–43]. In our research, we started the seeding density from 800 cells (low density) to 3200 cells (high density)/mm^2^. Based on the seeding density and the average cell size, the cell–cell proximity is 21 μm (low density), 9 μm (medium density), and 1 μm in the high-density seeding condition (Additional file 1: Figure S1c). Our results suggest that the microglia-glioma colonies could be formed in 24 h when both cells had enough proximity (at least in the medium-density seeding condition). Glioma cell lines were found to form colonies under specific culturing settings, including serum-free culturing, low-adherent conditions, or in a 3D environment. Interestingly, our study showed that glioma colonies could be formed in the presence of microglia without previously mentioned specific culturing conditions [44, 45]. To our knowledge, this is the first report demonstrating that microglia cells could render brain tumor cells to form a spheroid structure rapidly under normal cell culture conditions. The mechanism of the microglia-glioma colony formation is currently unknown and needs further clever investigation.

However, it is interesting that microglia-associated ALTS1C1 colonies were more resistant to the therapeutic drugs. Many studies have shown that tumor cells within a spheroid are more resistant to drugs than cells within a two-dimensional culture condition. The well-documented theories are that either cells within the spheroid have less proliferative or more stem cell-like features [46, 47]. The ALTS1C1 cells within microglia-associated colonies do not express stem cell marker CD133 or other stem cell-associated mRNA (data not shown) but are more cells in the G0/G1 phase, which may partially explain the resistance of these cells. Moreover, this study demonstrated that the administration of gap junction protein inhibitor, CBX, could disrupt colony formation and diminish the chemo-protective effect of microglia, indicating the vital role of cell–cell direct contact in microglia-mediated colony formation and drug resistance.

The cell–cell direct contact for exchanging cellular components via extracellular vesicles (EVs), cell fusion, and microtube networks has been suggested as important communication between cells [48–50]. The transfer of cellular components, including mRNA, nutrients, organelles, or drug substances, is dynamic and bi-directional between tumor cells and their surrounding stromal cells [50]. Studies have shown controversial results regarding the transfer direction from stromal cells to cancer or vice versa. For example, one study demonstrated that mitochondria were transferred from leukemia cancer cells to mesenchymal stem cells (MSCs) [15]. However, another similar study revealed that mitochondria were transferred from MSCs to the primary GBM-derived cells [51]. Mitochondria are considered subcellular organelles for chemo-drug accumulation; thus, moving direction will be important for the stromal cell-mediated tumor resistance or sensitivity to the chemo-drugs [52]. In this study, although we didn’t specifically focus on intracellular organelles, we did observe bi-directional drug transferring between microglia and glioma cells but with a different magnitude. 40% of microglia cells obtained doxorubicin from the drug-pretreated glioma cells, while less than 10% of glioma cells secured doxorubicin from the drug-pretreated microglia cells. A similar conclusion was supported by using membrane dye. More microglia have the expression of Fast-DiO obtained from the dye-pretreated glioma cells; on the contrary, glioma cells rarely get dye signals from the dye-pretreated microglia cells. Our transwell experiments further demonstrated that cell–cell direct contact was important for the drug transferring from glioma cells to the microglia cells. The above finding suggested that microglia can hijack drug substances from the glioma cells to increase the chemo-resistance of the glioma cells, and cell–cell contact was critical for drug transportation.

Resistance to the therapy hinders an effective treatment against glioma and leads to tumor recurrence; thus, overcoming the resistance may be key to a successful cure [10]. In our study, two directions to overthrow the resistance of glioma have been suggested. First, our results indicated that microglia played a vital role in affecting the resistance of the gliomas, and microglia were found to be the most affluent immune cells in the TME of glioma [19]. Therefore, microglia could be a promising target therapeutically. There are two main approaches to targeting microglia [20]. For example, using C–C chemokine 2 (CCL2) to block the recruitment of tumor-associated macrophages/microglia, or the colony-stimulating factor-1 (CSF-1R) inhibitor to impair the survival of macrophages/microglia [20]. Both molecules had promising results in the animal studies but did not meet expectations in the human clinical trials for compensation effects after the molecular blockade [53]. The reasons might be that the embargo didn’t fully deplete the targeted immune cell; instead, other subtypes of myeloid cells were increased and recruited to the tumor to restore the TME [54]. Whether different cells interact distinctively to affect the resistance of the tumor cells, more investigation on other immune or stromal cells remains to be determined. Reprogramming has been another way to target microglia by changing the microglia activation state from pro-tumor to anti-tumor via Toll-like receptors (TLR) or CD40 agonists [55]. Here, our study didn’t prove the alteration of the activation state of BV2 microglia; thus, whether the activation state of microglia affects the chemo-protection effect on the glioma cells needs to be further explored. Second, cell–cell interactions involve cells communicating facilitated by gap junction proteins, and connexins. Studies reveal that these proteins are often heightened in various cancers, playing crucial roles in growth, progression, and metastasis [56]. Munoz et al. discovered that epidermal growth factor receptor-induced connexin 43 led to chemoresistance in glioma cells against TMZ [57]. Another investigation focused on glioma cells and their neighboring astrocytes, revealing a link between intercellular communication via gap junctions, heightened chemo-resistance, and the activation of genes associated with survival pathways like mitogen-activated protein kinase and tyrosine-protein kinase [58]. Hence, targeting the gap junction proteins to block the cell–cell interaction seems promising. Our results have proved the feasibility of using gap junction protein inhibitor, CBX, to decrease microglia-associated drug resistance. Some in vivo animal research has also successfully shown that the administration of CBX could enhance the anti-tumor activity of chemo drugs [16, 37].

Several FDA-approved pan gap-junction inhibitors, such as 1-octanol, mefloquine, halothane, histamine, and CBX, find widespread application across unrelated conditions outside cancer therapy [59]. While we recognize the potential advantages of using CBX, it’s crucial to remain attentive to the potential for its broad inhibitory impact. This impact might extend beyond cancer cells, affecting normal cells and potentially resulting in side effects. Moreover, the CBX we’re utilizing is relatively non-specific, even though there are reports demonstrating its main inhibition effect on Cx43 [37]. It’s worth noting that CBX might also impact other connexin proteins or have non-gap junction-related effects, such as reducing seizures and enhancing cognitive function [60]. Therefore, using a more specific inhibitor could be a preferable approach. In our study, we have centered our focus on utilizing CBX as a broad gap junction inhibitor to evaluate whether the potential protective effect on glioma in BV2 cells comes from cell–cell interaction.

In our study, we’re utilizing the HSV-Tk suicide gene system to specifically target glioma cells. Although its cytotoxic mechanism is similar to other DNA-alkylating drugs including TMZ, and procarbazine [2], whether microglia cells could protect glioma cells from other therapeutics remained to be investigated. Moreover, our observation of chemo-substance transfer from glioma cells to microglia cells suggests the intriguing possibility that microglia cells could potentially safeguard glioma cells. From conventional ROS-accumulated radiation therapy to innovative approaches like oncolytic virus therapy, where tumor cells are overloaded with the virus to trigger apoptosis and subsequently the antitumor response from infiltrated immune cells [61, 62]. This intriguing avenue invites further investigation in not only the murine model but human or clinical model to the complex mutual interactions between microglia and glioma cells across a spectrum of therapeutic modalities. In summary, this study demonstrated that microglia could form colony structures with glioma cells. Glioma cells could escape from drug-induced apoptosis by communicating with microglia. Tumor-educated microglia could hijack drug substances from the glioma cells with direct contact and reduce the cytotoxic effect of therapeutic drugs. Blocking cell–cell interaction could impair these effects. This study elicits the role of microglia in brain tumor resistance and provides a novel target to improve the efficacy of brain tumor therapy.

### Supplementary Information

**Additional file 1: Figure S1.** (a) The immunofluorescence staining of whole murine orthotopic day-18 brain tumors, the nucleus was stained with Hoechst (blue), tumor cells (GFP), and the TMEM119 (Red) staining for microglia cells. Scale bar = 500 µm. (b) Larger immunofluorescence staining view of tumors (adjacent tissue section of Fig S1a). Scale bar = 200 µm. (c) The table of different co-culture seeding conditions in a 12-well dish. (d) Confocal image of ALTS1C1-GFP and BV2-Dil (shown in red, staining with dye Fast-DiL) co-culture, Blue arrow indicate the colony (left picture), and the magnified view of colony (right picture). Scale bar = 50 µm. (e) Co-culture of ALTS1C1 and BV2 under the condition medium from their co-culture, picture were taken after 24 h of culturing. Scale bar = 200 µm. **Figure S2.** Chemo-resistance of astrocytoma after co-culturing with BV2. (a) MTT assay of Cisplatin on ALTS1C1 and BV2 cell lines. (b) Experimental scheme of chemo-apoptosis assay and the flow cytometry gating strategy. (c) Representative dot plot FACs images of the co-culture chemo-apoptosis assay of 10 μg/mL  pro-drug GCV on BV2 only or ALTS1C1 co-cultured group for 0, 24, 36 hours of treatment. (d) MTT assay of pro-drug GCV on ALTS1C1-Tk and ALTS1C1-GFP-Tk cell line. (e) Representative caspase-3 staining images of BV2 treated with GCV for 24 hours. Scale bar =  200 μm. **Figure S3.** Chemo-resistance of GL261-Tk after co-culturing with BV2. (a) Representative dot plot FACs images of the co-culture chemo-apoptosis assay of 10 μg/mL pro-drug GCV on GL261-Tk only or GL261-Tk (+BV2) group for 36, 48, 60 hours. (b) Quantification of the chemo-apoptosis assay, A two-tailed unpaired t-test was used to compare every two groups. *: P <0.05, ****: P <0.0001. N≧6 for each group. **Figure S4.**. The cell cycle analysis on BV2 of different culturing conditions (Low density, High density, and co-cultured with ALTS1C1 on high density). A two-tailed unpaired t-test was used to compare every two groups. *: P <0.05, **: P <0.01.**Figure S5.**. BV2 hijack drugs from ALTS1C1 through direct contact. (a) Experimental scheme on Fast-DiO transfer test between the ALTS1C1 and BV2 cells. (b) Representative flow images of FITC-positive cell percentages indicating Fast-DiO-positive cells. (c) The quantification of Fast-DiO-positive cells after co-culturing for 12, and 24 hours. (d) The time-lapse video, Video link: https://www.youtube.com/watch?v=J9v2FsAB_bY. A two-tailed unpaired t-test was used to compare every two groups. **: P <0.01. **Figure S6.**. Annexin V/PI assay on the BV2 and ALTS1C1 cells (a) Representative dot plot FACs images of the chemo-apoptosis assay of 10 μg/mL  pro-drug GCV on BV2 or ALTS1C1 cells for 24 hours of treatment. (b) The statistics of the chemo-apoptosis assay. N>3 for each group.

**Additional file 2:** **Video S1**. The time-lapse video picturing Dox transferring from ALTS1C1-GFP to BV2. Images were taken every 5 minutes. The yellow arrow indicated the BV2 cell that obtained the dox from nearby ALTS1C1-GFP cells. Scale bar=25 μm.

## Data Availability

All data presented within the article and its supplementary information files are available upon request from the corresponding author.

## Abbreviations

TAMs: Tumor associated macrophages
CNS: Central nervous system
GBM: Glioblastoma
TMZ: Temozolomide
PFS-6: Progression-free survival rate at 6 months
HGG: High-grade (WHO classification; grade >III) glioma
TME: Tumor microenvironment
IL-6: Interleukin-6
STAT3: Signal transducer and activator of transcription 3
ROS: Reactive oxygen species
IACUC: Institutional Animal Care and Use Committee
HSV-Tk: Herpes simplex virus thymidine kinase
GCV: Ganciclovir
CBX: Carbenoxolone
Dox: Doxorubicin
MFI: Median fluorescence intensity
Cx43: Connexin 43
EVs: Extracellular vesicles
MSCs: Mesenchymal stem cells
CCL2: C–C chemokine 2
CSF-1R: Colony-stimulating factor-1
TLR: Toll-like receptors
PI: Propidium Iodide
