# Supplementary material for: Microglia-mediated drug substance transfer promotes chemoresistance in brain tumors: insights from an in vitro co-culture model using GCV/Tk prodrug system
Source: Cancer Cell Int. 2024 Jan 18;24:35. doi: 10.1186/s12935-024-03213-8 (PMC10795391; doi:10.1186/s12935-024-03213-8)
Supplement: Supplementary file 1 — Additional file 1: Figure S1. (a) The immunofluorescence staining of whole murine orthotopic day-18 brain tumors, the nucleus was stained with Hoechst (blue), tumor cells (GFP), and the TMEM119 (Red) staining for microglia cells. Scale bar = 500 µm. (b) Larger immunofluorescence staining view of tumors (adjacent tissue section of Fig S1a). Scale bar = 200 µm. (c) The table of different co-culture seeding conditions in a 12-well dish. (d) Confocal image of ALTS1C1-GFP and BV2-Dil (shown in red, staining with dye Fast-DiL) co-culture, Blue arrow indicate the colony (left picture), and the magnified view of colony (right picture). Scale bar = 50 µm. (e) Co-culture of ALTS1C1 and BV2 under the condition medium from their co-culture, picture were taken after 24 h of culturing. Scale bar = 200 µm. Figure S2. Chemo-resistance of astrocytoma after co-culturing with BV2. (a) MTT assay of Cisplatin on ALTS1C1 and BV2 cell lines. (b) Experimental scheme of chemo-apoptosis assay and the flow cytometry gating strategy. (c) Representative dot plot FACs images of the co-culture chemo-apoptosis assay of 10 μg/mL pro-drug GCV on BV2 only or ALTS1C1 co-cultured group for 0, 24, 36 hours of treatment. (d) MTT assay of pro-drug GCV on ALTS1C1-Tk and ALTS1C1-GFP-Tk cell line. (e) Representative caspase-3 staining images of BV2 treated with GCV for 24 hours. Scale bar = 200 μm. Figure S3. Chemo-resistance of GL261-Tk after co-culturing with BV2. (a) Representative dot plot FACs images of the co-culture chemo-apoptosis assay of 10 μg/mL pro-drug GCV on GL261-Tk only or GL261-Tk (+BV2) group for 36, 48, 60 hours. (b) Quantification of the chemo-apoptosis assay, A two-tailed unpaired t-test was used to compare every two groups. *: P <0.05, ****: P <0.0001. N≧6 for each group. Figure S4.. The cell cycle analysis on BV2 of different culturing conditions (Low density, High density, and co-cultured with ALTS1C1 on high density). A two-tailed unpaired t-test was used to compare every two groups. [file 12935_2024_3213_MOESM1_ESM.pptx]

## Slide 1
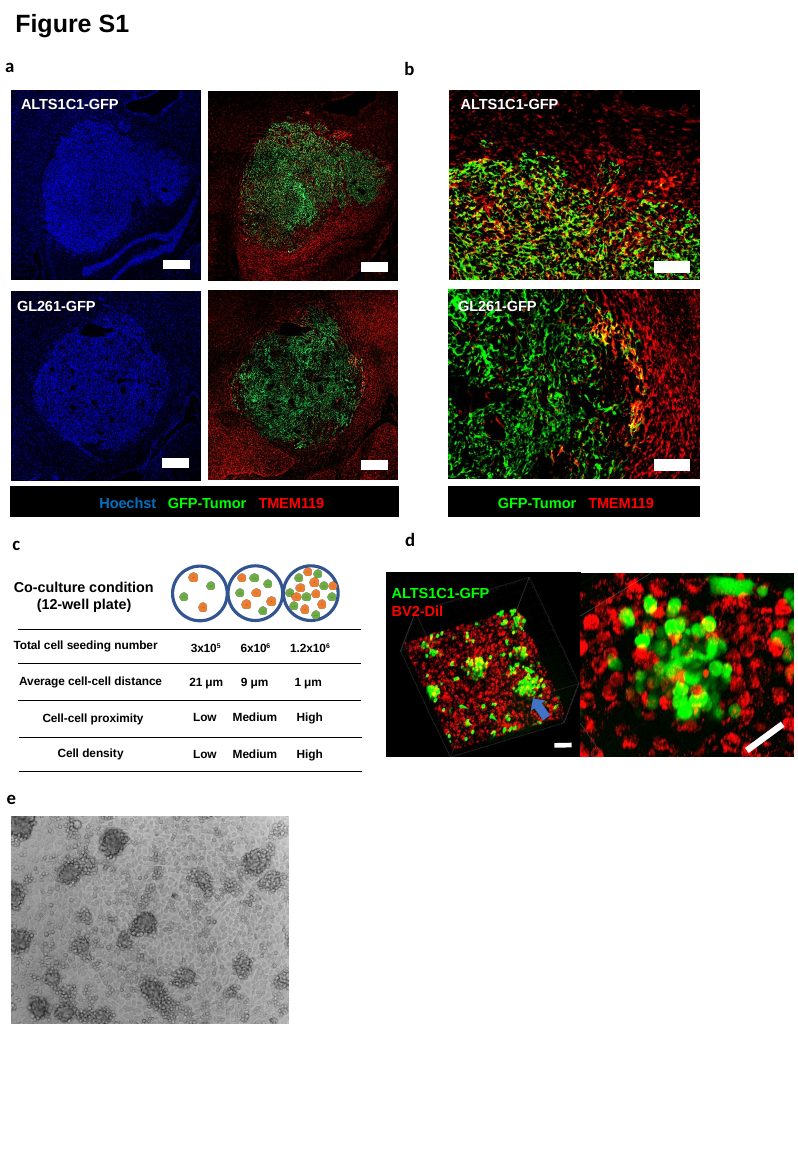

Figure S1
a
b
ALTS1C1-GFP
ALTS1C1-GFP
GL261-GFP
GL261-GFP
 GFP-Tumor TMEM119
Hoechst GFP-Tumor TMEM119
d
ALTS1C1-GFP
BV2-Dil
c
Co-culture condition
(12-well plate)
Total cell seeding number
3x10^5^ 6x10^6^ 1.2x10^6^
Average cell-cell distance
21 μm
9 μm
1 μm
Medium
Low
High
Cell-cell proximity
Cell density
Medium
High
Low
e

## Slide 2
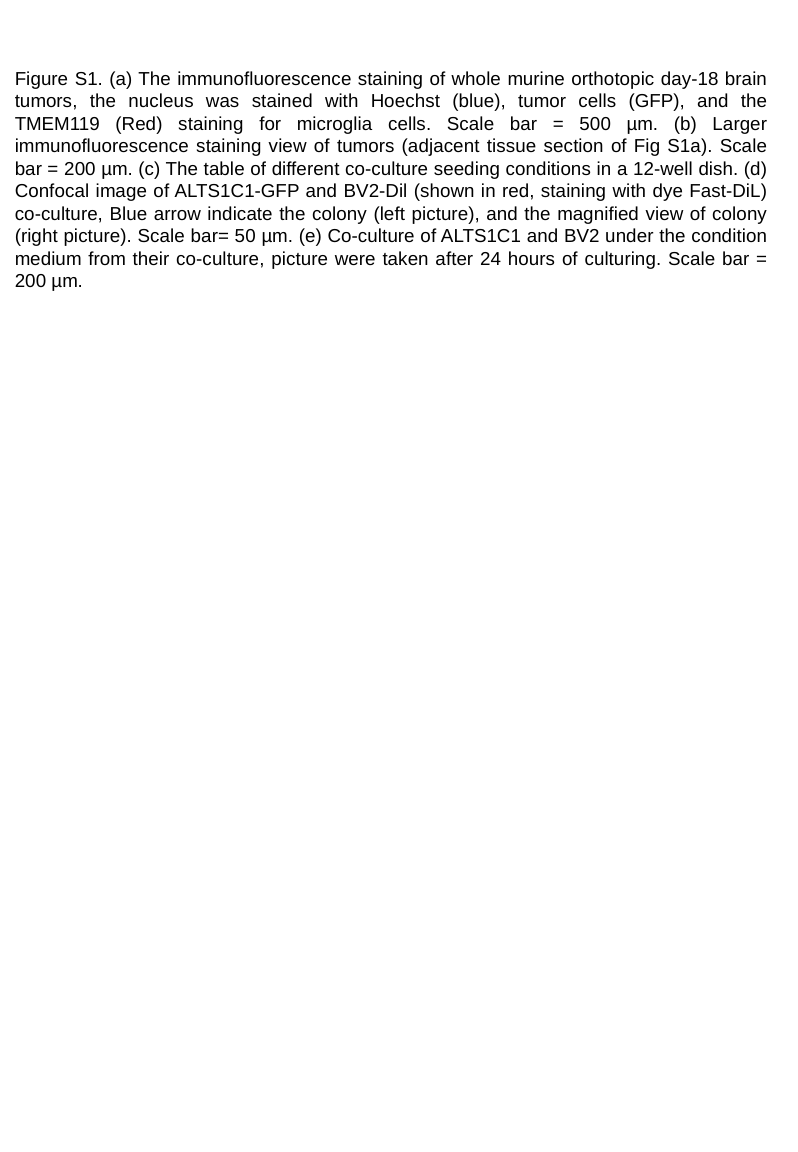

Figure S1. (a) The immunofluorescence staining of whole murine orthotopic day-18 brain tumors, the nucleus was stained with Hoechst (blue), tumor cells (GFP), and the TMEM119 (Red) staining for microglia cells. Scale bar = 500 µm. (b) Larger immunofluorescence staining view of tumors (adjacent tissue section of Fig S1a). Scale bar = 200 µm. (c) The table of different co-culture seeding conditions in a 12-well dish. (d) Confocal image of ALTS1C1-GFP and BV2-Dil (shown in red, staining with dye Fast-DiL) co-culture, Blue arrow indicate the colony (left picture), and the magnified view of colony (right picture). Scale bar= 50 µm. (e) Co-culture of ALTS1C1 and BV2 under the condition medium from their co-culture, picture were taken after 24 hours of culturing. Scale bar = 200 µm.

## Slide 3
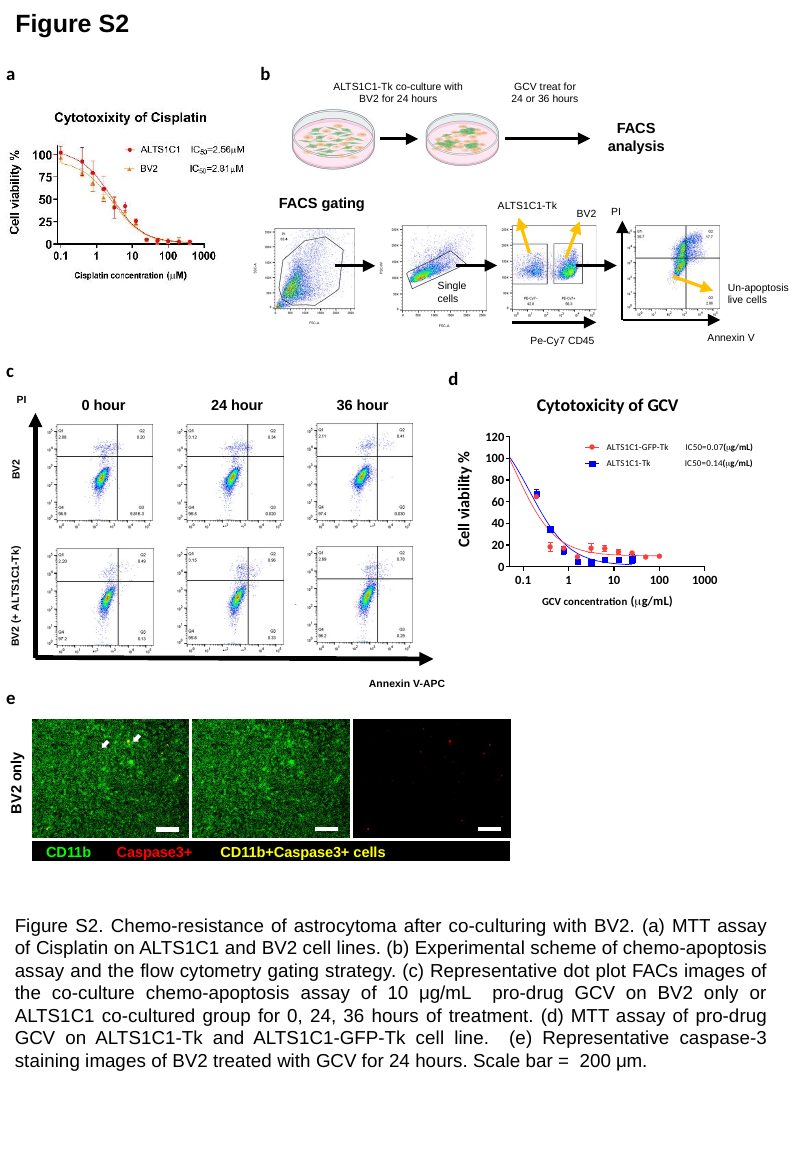

Figure S2
b
ALTS1C1-Tk co-culture with BV2 for 24 hours
GCV treat for 24 or 36 hours
FACS analysis
FACS gating
ALTS1C1-Tk
PI
BV2
Single cells
Un-apoptosis live cells
Annexin V
Pe-Cy7 CD45
a
c
PI
0 hour
24 hour
36 hour
BV2
BV2 (+ ALTS1C1-Tk)
Annexin V-APC
d
e
BV2 only
Caspase3+
CD11b
CD11b+Caspase3+ cells
Figure S2. Chemo-resistance of astrocytoma after co-culturing with BV2. (a) MTT assay of Cisplatin on ALTS1C1 and BV2 cell lines. (b) Experimental scheme of chemo-apoptosis assay and the flow cytometry gating strategy. (c) Representative dot plot FACs images of the co-culture chemo-apoptosis assay of 10 μg/mL pro-drug GCV on BV2 only or ALTS1C1 co-cultured group for 0, 24, 36 hours of treatment. (d) MTT assay of pro-drug GCV on ALTS1C1-Tk and ALTS1C1-GFP-Tk cell line. (e) Representative caspase-3 staining images of BV2 treated with GCV for 24 hours. Scale bar = 200 μm.

## Slide 4
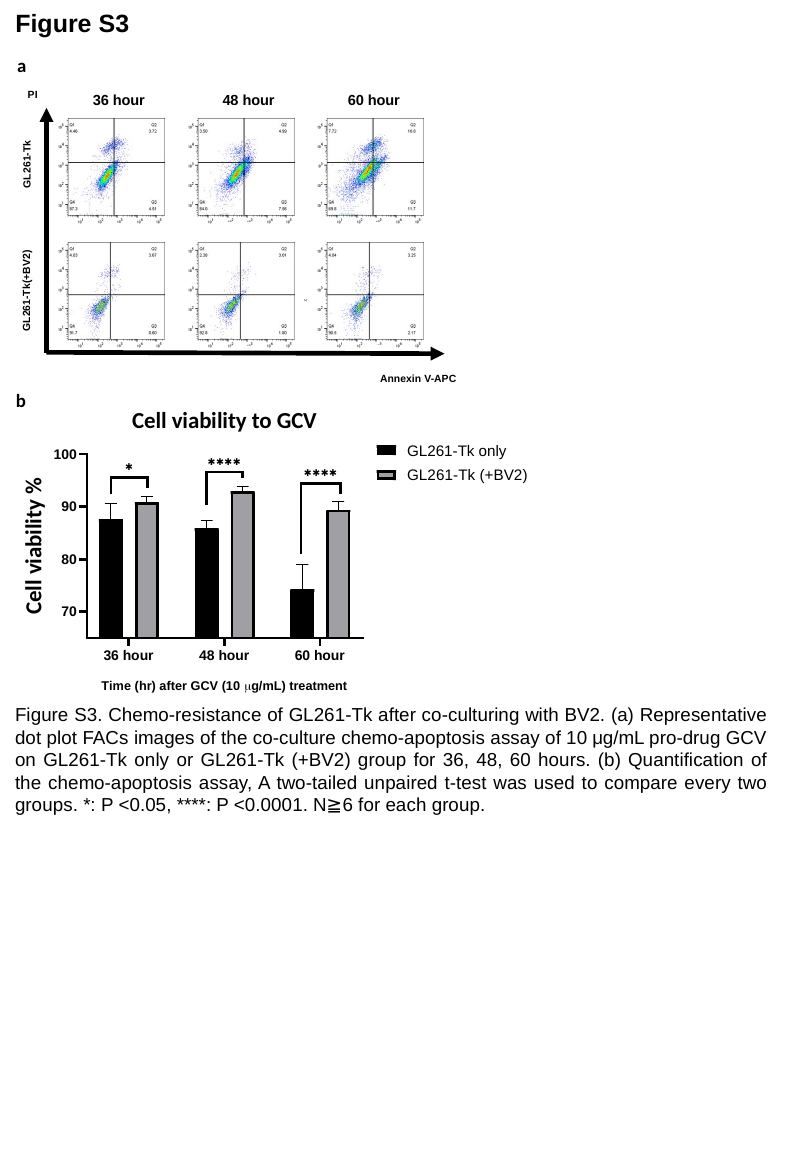

Figure S3
a
PI
36 hour
48 hour
60 hour
GL261-Tk
GL261-Tk(+BV2)
Annexin V-APC
b
Figure S3. Chemo-resistance of GL261-Tk after co-culturing with BV2. (a) Representative dot plot FACs images of the co-culture chemo-apoptosis assay of 10 μg/mL pro-drug GCV on GL261-Tk only or GL261-Tk (+BV2) group for 36, 48, 60 hours. (b) Quantification of the chemo-apoptosis assay, A two-tailed unpaired t-test was used to compare every two groups. *: P <0.05, ****: P <0.0001. N≧6 for each group.

## Slide 5
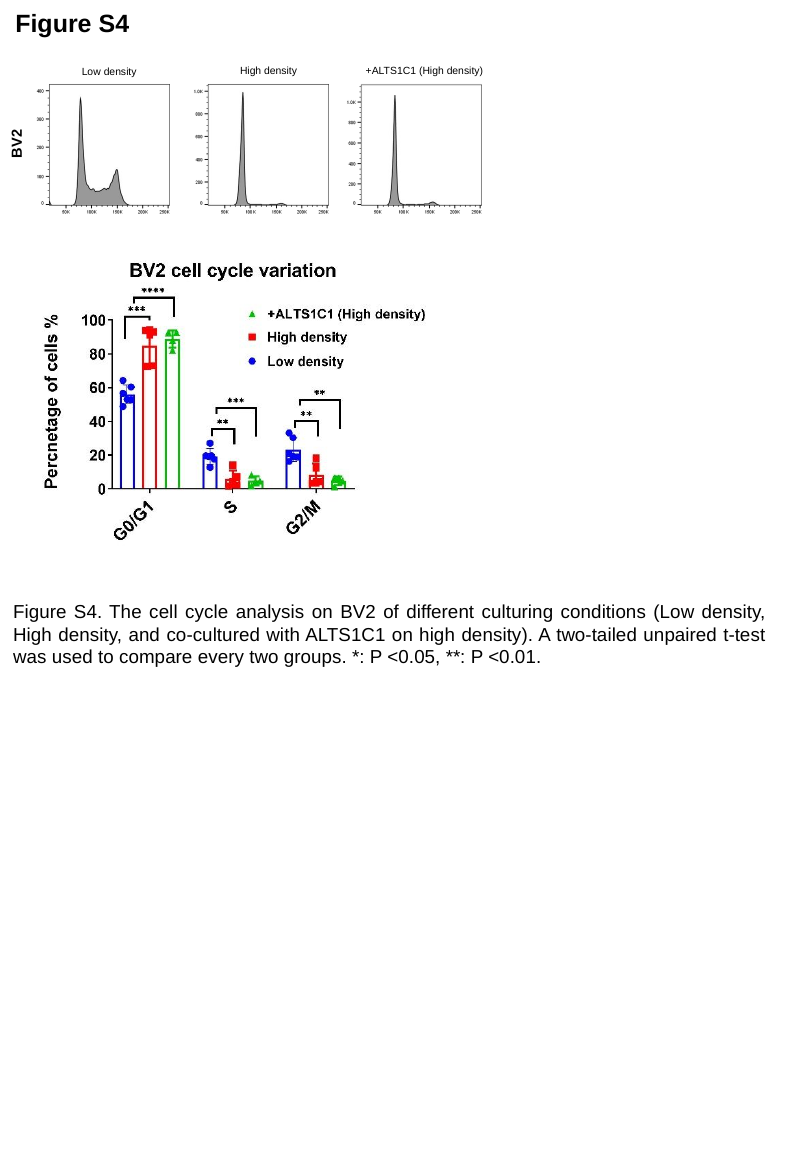

Figure S4
High density
+ALTS1C1 (High density)
Low density
BV2
Figure S4. The cell cycle analysis on BV2 of different culturing conditions (Low density, High density, and co-cultured with ALTS1C1 on high density). A two-tailed unpaired t-test was used to compare every two groups. *: P <0.05, **: P <0.01.

## Slide 6
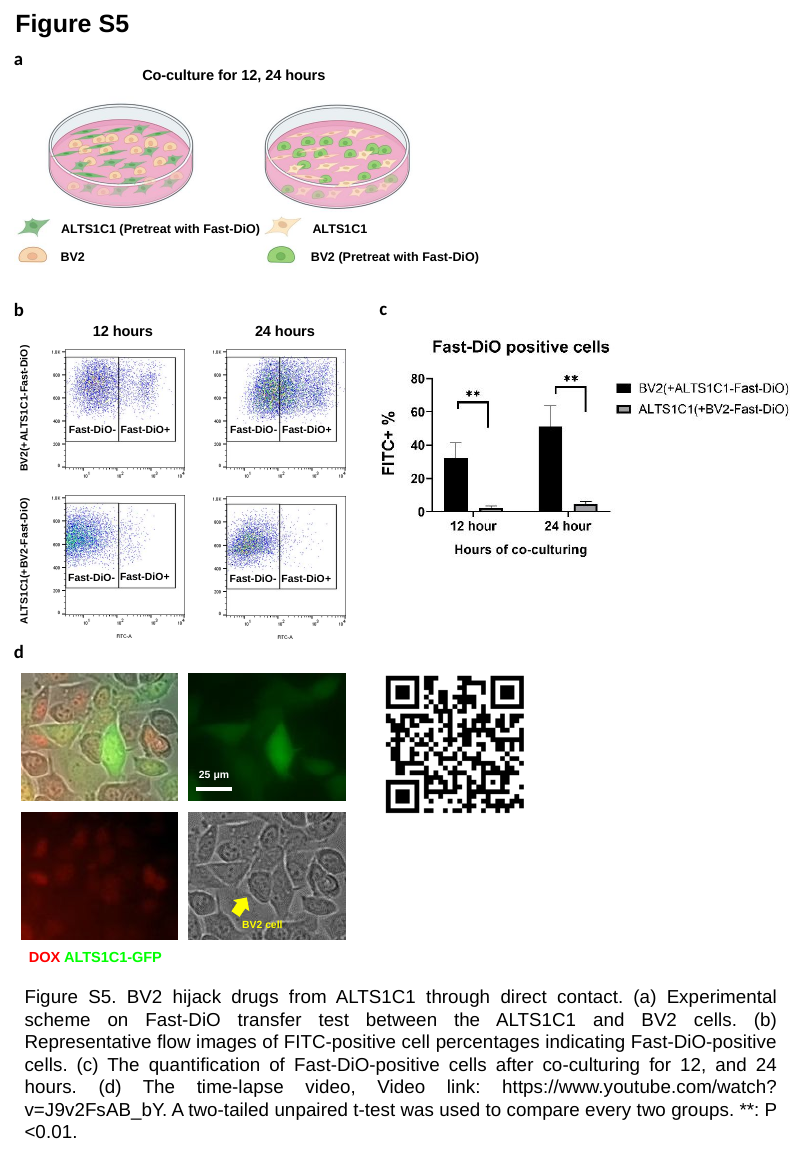

Figure S5
a
Co-culture for 12, 24 hours
ALTS1C1 (Pretreat with Fast-DiO)
ALTS1C1
BV2 (Pretreat with Fast-DiO)
BV2
c
b
12 hours
24 hours
BV2(+ALTS1C1-Fast-DiO)
Fast-DiO-
Fast-DiO-
Fast-DiO+
Fast-DiO+
ALTS1C1(+BV2-Fast-DiO)
Fast-DiO-
Fast-DiO-
Fast-DiO+
Fast-DiO+
d
25 μm
BV2 cell
DOX ALTS1C1-GFP
Figure S5. BV2 hijack drugs from ALTS1C1 through direct contact. (a) Experimental scheme on Fast-DiO transfer test between the ALTS1C1 and BV2 cells. (b) Representative flow images of FITC-positive cell percentages indicating Fast-DiO-positive cells. (c) The quantification of Fast-DiO-positive cells after co-culturing for 12, and 24 hours. (d) The time-lapse video, Video link: https://www.youtube.com/watch?v=J9v2FsAB_bY. A two-tailed unpaired t-test was used to compare every two groups. **: P <0.01.

## Slide 7
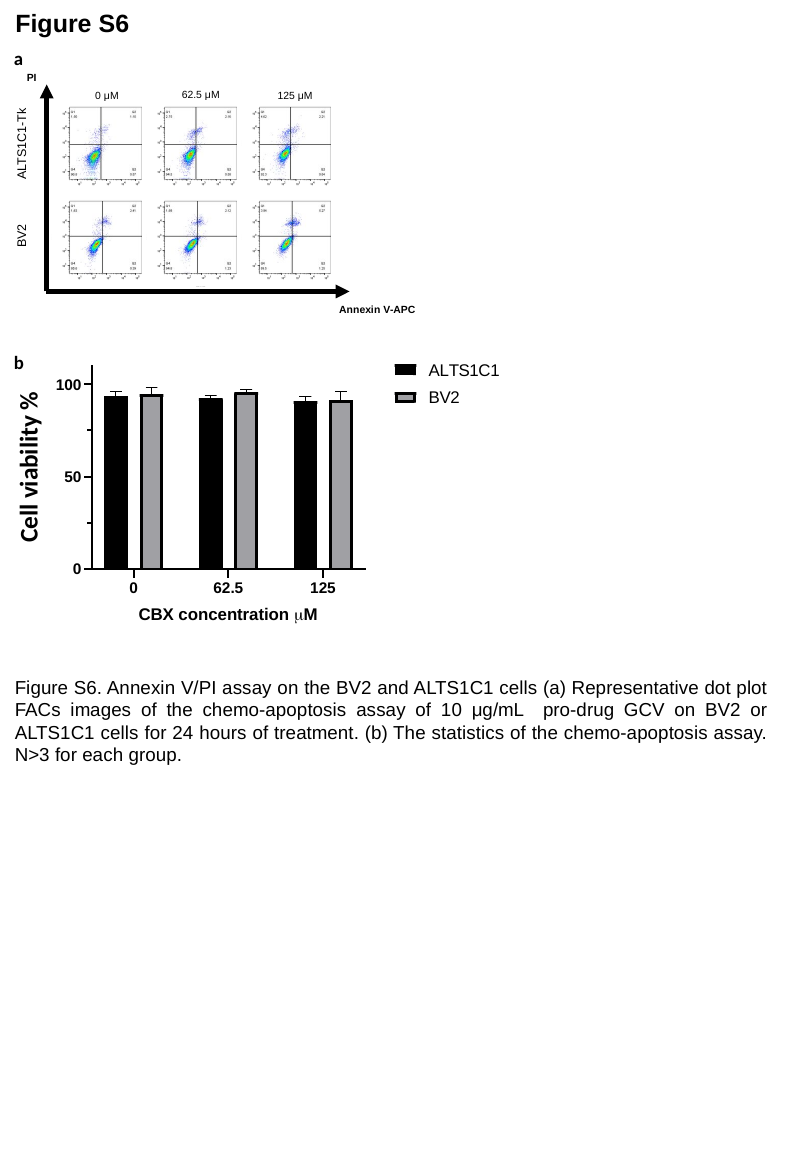

Figure S6
a
PI
62.5 μM
0 μM
125 μM
ALTS1C1-Tk
 BV2
Annexin V-APC
b
Figure S6. Annexin V/PI assay on the BV2 and ALTS1C1 cells (a) Representative dot plot FACs images of the chemo-apoptosis assay of 10 μg/mL pro-drug GCV on BV2 or ALTS1C1 cells for 24 hours of treatment. (b) The statistics of the chemo-apoptosis assay. N>3 for each group.
